# Validation of de-identified record linkage to ascertain hospital admissions in a cohort study

**DOI:** 10.1186/1471-2288-11-42

**Published:** 2011-04-08

**Authors:** Alison Beauchamp, Andrew M Tonkin, Helen Kelsall, Vijaya Sundararajan, Dallas R English, Lalitha Sundaresan, Rory Wolfe, Gavin Turrell, Graham G Giles, Anna Peeters

**Affiliations:** 1 Department of Epidemiology and Preventive Medicine, Monash University, Melbourne, Australia; 2 Victorian Data Linkages, Department of Health, Melbourne, Australia; 3 Department of Medicine, Monash Medical Centre, Monash University, Melbourne, Australia; 4 Cancer Epidemiology Centre, Cancer Council Victoria, Melbourne, Australia; 5 Centre for Molecular, Environmental, Genetic and Analytic Epidemiology, School of Population Health, University of Melbourne, Melbourne, Australia; 6 School of Public Health, Queensland University of Technology, Brisbane, Australia

## Abstract

**Background:**

Cohort studies can provide valuable evidence of cause and effect relationships but are subject to loss of participants over time, limiting the validity of findings. Computerised record linkage offers a passive and ongoing method of obtaining health outcomes from existing routinely collected data sources. However, the quality of record linkage is reliant upon the availability and accuracy of common identifying variables. We sought to develop and validate a method for linking a cohort study to a state-wide hospital admissions dataset with limited availability of unique identifying variables.

**Methods:**

A sample of 2000 participants from a cohort study (n = 41 514) was linked to a state-wide hospitalisations dataset in Victoria, Australia using the national health insurance (Medicare) number and demographic data as identifying variables. Availability of the health insurance number was limited in both datasets; therefore linkage was undertaken both with and without use of this number and agreement tested between both algorithms. Sensitivity was calculated for a sub-sample of 101 participants with a hospital admission confirmed by medical record review.

**Results:**

Of the 2000 study participants, 85% were found to have a record in the hospitalisations dataset when the national health insurance number and sex were used as linkage variables and 92% when demographic details only were used. When agreement between the two methods was tested the disagreement fraction was 9%, mainly due to "false positive" links when demographic details only were used. A final algorithm that used multiple combinations of identifying variables resulted in a match proportion of 87%. Sensitivity of this final linkage was 95%.

**Conclusions:**

High quality record linkage of cohort data with a hospitalisations dataset that has limited identifiers can be achieved using combinations of a national health insurance number and demographic data as identifying variables.

## Background

Cohort studies are a valuable source of information for epidemiological research, primarily because information about potential risk factors is collected before the outcomes of interest occur [1]. For example, the long-standing Framingham cohort study was critical in demonstrating the relationship between certain risk factors and the development of cardiovascular disease (CVD) events during follow-up [2-4].

The quality of evidence from cohort studies relies on complete and accurate ascertainment of outcomes such as myocardial infarction or stroke. Information about these and other health outcomes can be collected in a variety of ways, including medical record review and self-report from participants. While the former is considered the "gold standard" [5,6], it is particularly resource intensive for large cohorts. In addition, over longer periods of time, medical records may be difficult to locate or may be destroyed according to legislative requirements. Self-report from participants has been shown to have varying accuracy [5-10], and is subject to "loss to follow-up", an inherent problem and source of bias in cohort studies. Specific groups at risk of loss to follow-up include those of lower socioeconomic status and those with poorer health, often the groups of major interest to epidemiological research.

An alternative method of obtaining health outcome data for cohort studies is computerised record linkage [11,12]. This is the process of using common identifiers to link the cohort data with health services administrative or other datasets, for example to identify whether study participants have been admitted to hospital, and for which medical conditions [9,13]. Record linkage enables the optimal use of data from cohort studies because it permits "passive" follow-up, so that even if participants have been lost to follow-up from the study, information on specific health outcomes can still be obtained [11].

There are two main methods for computerised record linkage, probabilistic and deterministic. Probabilistic record linkage links records based on the statistical probability that common identifiers belong to the same person [11]. Deterministic linkage links two records based on complete agreement between the common identifiers [12]. Deterministic linkage is particularly suited to linkage of individual level data where accuracy is important and where data quality within the various datasets is high [14,15].

Linkage of cohort data with health services administrative datasets has been routine in several countries including the United Kingdom [13,16], Canada [17,18], and Sweden [19] for many years. It is important to note that for many of these studies, particularly those that use deterministic methods, linkage is based on unique identifiers such as social security number or health record number that are used by individuals throughout a lifetime and across the spectrum of health and social services [11,20,21]. However, it is not always possible to use unique identifiers, either because they are not fully available within the datasets, or because legislation requires that anonymity of records is maintained. In these situations, combinations of non-unique identifiers must be used such as date of birth, sex, and postal code. A key issue for researchers then becomes one of accuracy; that is, whether the combination of identifying variables used is sufficiently precise to identify the correct person, but not so broad as to incorrectly match to another person who has the same demographic data. In addition, it is essential to make allowance for mistakes in data entry particularly when using deterministic methods of linkage in which records are linked only if they match exactly [22]. Therefore, when developing a method for linking two or more datasets with limited identifying information, validation of the linkage is vital.

We planned to link a cohort study with a state-wide hospital admissions dataset in order to obtain data on incident CVD events occurring in the cohort during 19 years of follow-up. The hospitalisations dataset did not contain names and addresses, and there was limited availability of unique identifiers in either dataset. In this study, we therefore sought to determine, in a subgroup of the cohort, the most accurate combinations of identifying variables with and without use of the national health insurance number, with the overall aim of establishing a stepwise deterministic algorithm for linking the two datasets. We also aimed to test the sensitivity of the linkage for correctly identifying that a true hospital admission event had occurred.

## Methods

### Data Sources

The Victorian Admitted Episodes Dataset (VAED) is held by the Victorian Department of Health (DH), and includes information on all private and public hospital admissions in Victoria. While full names and addresses are not included in the dataset, approximately 81% of records in the VAED include Medicare card number, the national health insurance number allocated to all Australians. Medicare card numbers are unique to a family only and individual family members are identified by the Medicare suffix, comprising the first three letters of their given (first) name. The VAED is episode of care based, and the DH has linked these episodes using identifiers such as hospital record number (if episodes occurred in the same hospital) and the first 8 digits of Medicare card number, as well as identifiers such as date of birth, gender, postal code, country of birth and the first three digits of first or middle name (if episodes occurred in different hospitals). The methods for this internal linkage have been described elsewhere [23]. Data between 31 July 1996 and 31 December 2008 from this "internally linked VAED" were used for the current study. The VAED has previously been linked to a number of different datasets and registries including a transport accident dataset [22], a cardiac rehabilitation dataset [24], and a cardiothoracic elective surgery information system [25].

The Melbourne Collaborative Cohort Study (MCCS) is a prospective study of 41 514 subjects aged 27 to 80 years, recruited between 1990 and 1994. Details of the design, recruitment, and study procedures have been published elsewhere [26]. In brief, subjects were volunteers from metropolitan Melbourne, recruited using electoral rolls, community centres and churches. Between 2003 and 2008, attempts were made to re-interview all surviving participants; 28 240 participants were re-interviewed. At this interview, participants were asked to provide their Medicare card number and details on hospitalisations for cardiovascular and other diseases. This linkage study was undertaken on a random sample of 2000 participants who had been re-interviewed. The entire sample had Medicare details available. In addition, the sample was stratified to include 67% with a self-reported hospital admission for a cardiovascular event at re-interview to ensure at least this number of participants with a record in the VAED. CVD was chosen as the event of interest to allow us to test the sensitivity of linkage using a clearly defined outcome.

The pilot sample of 2000 used in this study included 101 participants with a confirmed hospital admission for myocardial infarction (AMI) or stroke between 1 July 1996 and the time of their re-interview. These 101 participants were used to test the sensitivity of linkage, with hospitalisation and diagnosis confirmed as follows: At re-interview all participants were asked "Has a doctor or nurse ever told you that you had a heart attack or myocardial infarction or stroke?" Hospital name and year of admission were also asked. From those who responded in the affirmative and had not reported a prior history of CVD at study baseline, a random sample of 400 participants was selected. We excluded 193 of these for the following reasons: hospital name not given or no medical records identified for that participant (n = 84), hospital medical records destroyed (n = 67), interstate hospitalisation (n = 21), and no CVD event identified in the medical record (n = 21). Data considered to relate to the self-reported CVD event was obtained from the medical records for 207 participants and coded by expert panels of neurologists and cardiologists. From the original sample of 400, we identified 124 participants with a confirmed admission for AMI or stroke occurring between baseline and the time of their re-interview. We excluded a further 23 of these with an event prior to 1 July 1996 (the commencement of the VAED), leaving a total of 101 participants with a confirmed AMI or stroke. These 101 were included in the pilot sample of 2000, flagged as "confirmed admission.'

The study protocol was approved by Human Research Ethics Committees at Monash University, The Cancer Council Victoria and the Victorian Department of Health. All subjects provided written informed consent, including for linkage to the VAED.

### Identifying variables used for linkage

Identifiers common to both the study sample and the VAED were the national health insurance (Medicare) card number and suffix (i.e., a given or middle name abbreviated to first 3 letters), date of birth, postcode, sex and country of birth. For linkage purposes, the first 8 numbers of the Medicare card number only were used (Medicare8). The above identifiers were joined to create a single linkage variable which was then encrypted.

### Linkage Method

Medicare card number is not fully available in either the VAED or the MCCS. We therefore undertook linkage using two algorithms in order to assess the effect of missing Medicare numbers. The first algorithm used combinations of Medicare and demographic details as linkage variables, and the second used demographic details only. For each algorithm, identifiers from each dataset were grouped into several combinations and matched in a stepwise deterministic strategy, using multiple iterations. Matches in each iteration were accepted only if the identifiers were identical between the two datasets. Records that matched in each iteration were removed from the source datasets for subsequent iterations.

### Agreement between linkage with and without Medicare number

Agreement between the two linkage algorithms (i.e. Medicare card number plus demographic details versus demographic details only) was assessed based on the assumption that if the two algorithms worked equally well, then each would link MCCS participants to the same record in the VAED.

### Sensitivity of linkage - Participants with confirmed admissions for AMI or stroke

The sensitivity of the linkage process was assessed using the 101 MCCS participants with a 'confirmed admission' for AMI or stroke. Information was extracted from the VAED relating to any hospital admissions they may have had between 1 July 1996 and 31 December 2008. If a participant's confirmed hospitalisation matched a record from the VAED by hospital name, dates of admission and discharge (within ten days), then the episode was considered to have been correctly identified by the linkage.

Sensitivity was calculated as the number of confirmed admissions that were correctly identified in the VAED divided by the total number of confirmed admissions.

## Results

### Availability of identifying variables

Medicare details were available for 100% of the study sample, and 80% of the VAED records. There was 100% availability of demographic variables from both datasets.

### Linkage Method

Tables 1 and 2 describe the combinations of linkage variables used and the number of study participants matched during each iteration, both with and without use of Medicare card number. When the Medicare number was used, 1865 of the 2000 (93%) records were matched to a record in the VAED (Table 1). The first 4 iterations of this algorithm used various combinations of Medicare number, suffix and sex only, and resulted in 1702 (85%) matches between the study sample and the VAED. When the Medicare number was not used, combinations of date of birth, sex, country of birth and postcode yielded matches for 1843 records (92%) (Table 2).

**Table 1:** Linkage using Medicare number and suffix (V1)

Iteration	Linkage Variables used in V1	Records Linked	Records remaining
1	Medicare8^1 ^+ Medsuf1^2^	1,667	333
2	Medicare8+first and second letters of Medsuf1+Sex	7	326
3	Medicare8+ second and third letters of Medsuf1+Sex	2	324
4	Medicare8+first and third letters of Medsuf1+Sex	0	324
5	Medicare8+Yearbirth+Sex	26	298
6	Medsuf1+Yearbirth+Monthbirth+Daybirth+Sex+Country of Birth+Postcode1	32	266
7	Medsuf1+Yearbirth+Monthbirth+Daybirth+Sex+Country of Birth+Postcode2	4	262
8	Medsuf1+Yearbirth+Monthbirth+Daybirth+Sex+Country of Birth+Postcode3	2	260
9	Medsuf1+Yearbirth+Monthbirth+Daybirth+Sex+Country of Birth+Postcode4	1	259
10	Yearbirth+Monthbirth+Daybirth+Sex+Country of Birth+Postcode1	124	135
	**Total Matched**	**1865**	**135**

^1 ^First 8 digits of the Medicare number; ^2 ^First 3 letters of the first name

**Table 2:** Linkage not using Medicare number and suffix (V2)

Iteration	Linkage Variables used in V2	Records Linked		Records remaining
1	Yearbirth+Monthbirth+Daybirth+Sex+Country of Birth+Postcode1	1,599		410
2	Yearbirth+Monthbirth+Daybirth+Sex+Country of Birth+Postcode2	193		208
3	Yearbirth+Monthbirth+Daybirth+Sex+Country of Birth+Postcode3	39		169
4	Yearbirth+Monthbirth+Daybirth+Sex+Country of Birth+Postcode4	12		157
	**Total Matched**	**1843**		**157**

### Agreement between linkage with and without Medicare number

**Figure 1:**
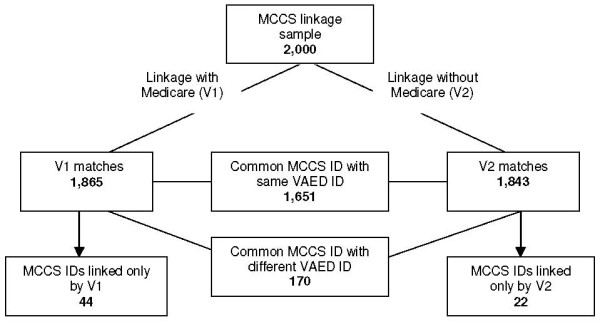
**Agreement between linkage with and without Medicare number**.

The two linkage methods matched 1651 MCCS records to records with the same VAED linkage ID (the computer generated number given to groups of hospitalisations thought to belong to the same individual as a result of the linkage process) (Figure 1). Another 170 MCCS participants were matched to different VAED IDs, implying that the two linkage methods had matched these participants to different records in the VAED dataset. This represents an 8.5% disagreement fraction. When the Medicare card number was not used (demographic variables only), all 170 records in each dataset matched exactly for every linkage variable. When the Medicare number and demographic details were used, 128 of the 170 matched exactly for every linkage variable. Assuming that these 128 matches were correct (as they had matched completely on the most distinctive combination of variables available), it is likely that at least 128 of the 170 from the linkage without Medicare numbers were 'false positive' matches, due to there being more than one person with a VAED record from the same postcode, born on the same date, and of the same sex and country of birth. Of the 42 remaining records identified, all matched on Medicare number and suffix. Most differences were seen in date of birth, which varied between 2-10 days and up to 40 years, suggesting possible errors in data entry or that the MCCS participant had been matched to another family member whose given name began with the same three letters.

There were 44 MCCS participants who were matched only in V1 (Figure 1). All of these matched completely on Medicare details, but all had at least one demographic variable that was unmatched; the most likely explanation for not being matched in V2. There were 22 MCCS participants who were matched only in V2. This was possibly because their Medicare card number had either been entered into the VAED or MCCS datasets incorrectly, or they did not have a Medicare card number in the VAED.

### Linkage without Medicare - decreasing the number of 'mismatches'

We sought to decrease the 8.5% disagreement fraction between linkage with and without Medicare details by adding the Medicare suffix to the linkage algorithm containing demographic variables only. This field is available for approximately 80% of records in the VAED and all MCCS participants. Applying this algorithm, 1633 out of 2000 (82%) records were matched. When compared with the linkage involving Medicare card number and demographic variables, there were 1620 participants who had the same VAED ID. Only 13 records, compared with 170 shown in Figure 1, had different VAED IDs on the two linkages. There were fewer matches than for the linkage based on demographic variables only (1633 compared to 1843), suggesting that adding the first three letters of the given name reduced the number of "false positives".

### Final algorithm

Based on the above findings, we developed a final algorithm that will be used to link the entire study cohort of 41 514 participants (Table 3). The algorithm is grouped into three stages. The first stage (Medicare card number and Medicare suffix) uses combinations of the national health insurance card number and first 3 letters of the first name plus all demographic details. The first iteration in this stage is assumed to provide the most correct match possible. Subsequent iterations of this first stage use variations of the Medicare suffix and also drop one variable at a time to allow for errors in data entry, with a maximum of variation in two variables allowed at one time. The second stage (Medicare card number) is used for matching records with variations in first names, for example where nicknames or middle names have been used. The final stage (Medicare suffix only) aims to match those records that do not have Medicare card number available in one or more dataset. Again, this group allows for variation in no more than two variables at a time. Running this algorithm resulted in 1740 (87%) of 2000 records linked.

**Table 3:** Suggested linkage cycles/iterations

	Linkage Variables	Records linked	Records remaining
**Iteration**	**Medicare8**^**1 **^**+ Medsuf1**^**2 **^**group**		
1-4	Medicare8+Medsuf1+Yearbirth+Monthbirth+Daybirth+Sex+COB+ Postcodes1-4	1594	406
5-16	Repeat steps 1-4 using 3 different variations of Medsuf1	8	398
17	Repeat step 1 dropping postcode	23	375
18-21	Repeat steps 1-4 dropping COB	7	368
22-25	Repeat steps 1-4 dropping sex	1	367
26-29	Repeat steps 1-4 dropping day of birth	19	348
30-33	Repeat steps 1-4 dropping month of birth	2	346
34-37	Repeat steps 1-4 dropping year of birth	17	329
38-100	Repeat steps 5-16, dropping 1 variable	0	329
101-141	Repeat steps 1-4 dropping two demographic variables	4	325
	**Medicare8 group**		
142-145	Medicare8+Yearbirth+Monthbirth+Daybirth+Sex+COB+ Postcodes 1-4	24	301
146-166	Repeat steps 142-145 dropping 1 variable	2	299
	**Medsuf1 Group**		
167-170	Medsuf1+Yearbirth+Monthbirth+Daybirth+Sex+COB+ Postcodes 1-4	39	260
**Total records linked = 1740/2000 = 87%**			

^1 ^First 8 digits of the Medicare number; ^2 ^First 3 letters of the first name

### Testing sensitivity - MCCS participants with 'confirmed admission'

We tested sensitivity using the sample of 101 participants with a confirmed hospital admission for AMI or stroke. In linkage undertaken using the final algorithm, 98 of these 101 (97%) participants were linked to a record in the VAED (Table 4). Of the 98 who were linked, the data we had obtained from the hospital medical record did not match the VAED data for admission and discharge dates and hospital name in 4 cases. Overall, this represents a sensitivity of 93% (94/101; 95% confidence interval 86% to 97%) for the VAED to correctly identify that a hospital admission for AMI or stroke has occurred when linkage was undertaken using the final algorithm.

**Table 4:** Number of VAED matches to MCCS participants with confirmed CVD event

	Linkage using Medicare details (V1)
Number of true CVD events (medical record review)	101
Number of admissions identified by VAED using NEWID	98
Number of CVD events correctly identified by VAED using NEWID	94
Sensitivity	94/101 = 93%
Number of incorrect matches in VAED	4
Non-matched rate	4/101 = 4%

## Discussion

### Overall findings

This study aimed to develop the most appropriate method for linking a large cohort study to a state-wide hospital admissions dataset, with limited availability of unique identifying variables in either dataset. We found that linkage using demographic variables only had the potential to create "false positive" links, which were reduced by adding the first three letters of the given (first) name to the linkage variables. The number of records linked was highest when using combinations of the national health insurance number, the first three letters of the first name, date of birth, sex, country of birth and postcode. Accordingly, we have developed a stepwise algorithm using combinations of identifying variables that will provide the greatest accuracy in deterministic linkage of cohorts such as the MCCS to administrative health datasets. Sensitivity of this linkage algorithm to correctly identify that a hospital admission had occurred was 93%.

### Limited identifying variables and advantages of Unique Personal Health Identifiers

This study addresses an important aspect of record linkage, that is how to link without using names and addresses or unique health identifiers. Our findings are relevant for custodians of existing research or administrative datasets who seek to increase their value through record linkage but do not have access to such identifying information. In the future, it is likely that record linkage with hospital and health administration datasets will be much enhanced by use of a Unique Personal Health Identifier (UPHI). These electronic numbers will be used to uniquely identify healthcare providers and individuals, and aim to both improve communication between health care providers and support the delivery of health services thus enhancing the quality of patient care [27-29]. UPHI's have also been advocated as a way of accurately linking records in a privacy preserving way [30]. While not currently used in Australia, legislation was recently passed authorising the issue of individual identifier numbers. However, it will be some time before uptake of the UPHI is widespread enough to allow for its use in linkage of health records, and for some existing datasets, this will never be possible. As such, for those datasets with limited identifying variables, our study demonstrates that record linkage to other datasets is achievable, further increasing their importance as a valuable source of health-related data.

### Effect of errors in data entry

While use of a UPHI or other unique identifier in combination with demographic details is likely to provide the most accurate linkage for health-related datasets, they may be subject to data entry errors. We found for example, that errors for day of birth ranged from between 2 to 10 days. We were unable to quantify the impact of data entry error in our study. However, others have found that such errors can be significant [20,31]. One study that linked the New York State AIDS registry and a hospital discharge file using date of birth, sex, admission dates and hospital record number found 82-85% accuracy, assessed by medical record review and manual verification [31]. In that study, errors in data entry accounted for most of the missed links. A study conducted in Indiana linking hospital admissions registries with a death registry using social security number (SSN) also found that errors in SSN were significant [20]. It may be possible for researchers using administrative or data sources to reduce the impact of data entry error by varying the combinations of variables used, as shown in our final algorithm. In addition, errors could be minimised by performing regular audits of data quality.

### Effect of missing linkage data

Both when UPHIs, names and addresses or when only demographic details are used as linkage variables, the degree of missing data must first be quantified and its impact reduced by creating linkage algorithms that allow for missing data. Our final linkage algorithm allows for missing health insurance numbers in either dataset by including abbreviated first name in addition to demographic variables. However, for current or ongoing data collections, researchers and data custodians will benefit from ensuring practices and protocols are in place to minimise the risk of missing data.

### Limitations

This study did not assess specificity, which is the ability of linkage to show that participants with no hospitalisations are correctly identified as such, or that participants are not linked to the wrong record. We were also unable to assess the validity of the 'linked VAED'. This dataset had previously been internally linked using Medicare card numbers, hospital record numbers or demographic data. Of the 101 'confirmed admissions', 3 did not link to a record in the VAED, indicating either errors in data entry or errors in the 'linked VAED'. However, the 'linked VAED' has previously been shown to be accurate [23], and sensitivity of linkage to correctly identify a hospital admission was relatively high at 96%, indicating that it is accurate for at least hospital name and dates of admission. A further limitation is that the Medicare card number may alter with change in family circumstances, such as divorce or marriage. The number of participants in the sample in whom this occurred is unknown, although is likely to be small given the high number of matches using Medicare number.

We found that performing linkage in which the national health insurance card number and abbreviated given name were "missing" from the study data increased the probability of matching to the wrong record in the hospital administrative dataset. This may lead to misclassification of health outcomes, likely to be non-differential in nature as the reason for misclassification will generally be independent of the exposure. While this non-differential disease misclassification may have implications for subsequent analyses using health outcomes data obtained from linkage, it is most likely to bias associations between exposures and outcomes towards the null [32].

### Implications

This pilot study has significantly increased the potential of a cohort study to determine health outcomes related to hospitalisation, even with limited availability of unique identifiers. The methods described may therefore be applicable to other settings in which linkage is undertaken using limited identifiers.

## Conclusion

Our findings suggest that record linkage with a hospital admissions dataset that has limited identifiers offers an opportunity to identify long-term health outcomes in an established cohort study, significantly increasing the value of the study. There are specific issues which affect the quality of linkage, and may have implications for use of data obtained from linkage. In the future, including Unique Personal Health Identifiers in administrative datasets used for record linkage would significantly improve the quality of such a valuable research tool.

## Competing interests

The authors declare that they have no competing interests.

## Authors' contributions

AB drafted the manuscript, participated in the study design, manually reviewed outcomes of linkage, undertook medical record review, and performed statistical analysis. AT participated in the study design and critically reviewed the manuscript. HK participated in the study design, assisted with interpretation of results and helped to draft the manuscript. VS assisted with the development of the linkage protocol, assisted with interpretation of results and helped to draft the manuscript. DE is an original investigator on the MCCS, participated in the study design and critically reviewed the manuscript. LS assisted with the development of the linkage protocol and undertook the linkage. RW assisted with statistical analysis and critically reviewed the manuscript. GT participated in the study design and critically reviewed the manuscript. GG DE is an original investigator on the MCCS and critically reviewed the manuscript. AP participated in the study design, assisted with interpretation of results and helped to draft the manuscript. All authors read and approved the final manuscript.

## Pre-publication history

The pre-publication history for this paper can be accessed here:

http://www.biomedcentral.com/1471-2288/11/42/prepub
